# Simultaneous genome sequencing of symbionts and their hosts

**DOI:** 10.1007/s13199-012-0154-6

**Published:** 2012-02-15

**Authors:** Sujai Kumar, Mark L. Blaxter

**Affiliations:** Institute of Evolutionary Biology, University of Edinburgh, Ashworth Laboratories, Edinburgh, EH9 3JT UK

**Keywords:** Symbiont, Second-generation sequencing, Genome, Nematode, Illumina

## Abstract

Second-generation sequencing has made possible the sequencing of genomes of interest for even small research groups. However, obtaining separate clean cultures and clonal or inbred samples of metazoan hosts and their bacterial symbionts is often difficult. We present a computational pipeline for separating metazoan and bacterial DNA in silico rather than at the bench. The method relies on the generation of deep coverage of all the genomes in a mixed sample using Illumina short-read sequencing technology, and using aggregate properties of the different genomes to identify read sets belonging to each. This inexpensive and rapid approach has been used to sequence several nematode genomes and their bacterial endosymbionts in the last year in our laboratory and can also be used to visualize and identify unexpected contaminants (or possible symbionts) in genomic DNA samples. We hope that this method will enable researchers studying symbiotic systems to move from gene-centric to genome-centric approaches.

## Introduction

Second-generation technologies such as Illumina, 454, SOLiD, and Helicos have reduced the costs of DNA sequencing and democratized genome projects (Fuller et al. 2009). Now, even small research groups can take genome-centric instead of gene-centric approaches to studying metazoan-bacterial symbiosis. Armed with complete gene catalogues for both the host and symbionts, we can study the metabolic pathways contributed by each organism (Moran and Mira 2001), the genes implicated in the evolution of symbiosis (Sinkins et al. 2005), lateral gene transfers between bacteria and metazoa (Dunning Hotopp et al. 2007) and symbiont-killing drug targets in the case of pathogenic hosts (Pfarr and Hoerauf 2006; Slatko et al. 2010).

*Wolbachia* are common symbionts of arthropod and nematode hosts, have significant ecological impact, and may be important for biocontrol and the treatment of parasitic diseases. Nine *Wolbachia* genomes have been completely or partially sequenced since 2002. The first *Wolbachia* genomes to be sequenced were derived from enriched *Wolbachia* DNA purified using pulsed field gel electrophoresis (Wu et al. 2004) or selected from a Bacterial Artificial Chromosome (BAC) library of BACs that hybridized to specific *Wolbachia* genes (Foster et al. 2005). However, physically separating endosymbionts (and their DNA) from the host is labor intensive and time consuming, and does not guarantee that the symbiont DNA will be free of host DNA: the process is best viewed as an enrichment step. In the case of *Wolbachia* from filarial parasitic nematodes, one has to contend with mammalian (3 gigabases (Gb)) and nematode (100 megabases (Mb)) genomes contaminating the 1 Mb *Wolbachia* genome. In such cases, even a few host nuclei can contribute most of the DNA in a preparation. Here we describe the use of Illumina short-read sequencing to completely sequence the bacterial *Wolbachia* endosymbiont of the filarial nematode *Dirofilaria immitis* (the dog heartworm) without prior purification of the symbiont from the host. We sequenced both organisms simultaneously and separated the DNA of the two species in silico using a combination of sequencing coverage information, DNA base composition, and sequence similarity searches.

Simultaneous sequencing of a host and its symbionts is not a new idea, though doing this deliberately is a departure from normal practice. Salzberg et al. (2005) extracted *Wolbachia* genomes ‘contaminating’ *Drosophila* genome sequencing projects by identifying raw sequence reads that had significant identity to the previously sequenced *Drosophila melanogaster Wolbachia* strain, *w*Mel. It is thus possible, given significant existing genomic information for both host and symbiont, to separate their genomes using computational rather than laboratory tools, but this richness of background data is not available for most symbioses. We thus sought to develop a method that could be used in situations where the genome sequences of both host and symbiont were unknown, or at least sufficiently divergent from the nearest sequenced comparators as to render the direct mapping of raw data to a reference genome ineffective or misleading. The problem of separating mixed taxa in a sequenced sample has been addressed extensively in the field of metagenome sequencing (Wooley et al. 2010). However, unlike most metagenomics studies where the goal is to sample and classify a diverse microbial community with tens to hundreds of bacterial taxa, our goal was to obtain a complete draft genome for an individual metazoan host and its endosymbiont.

Aggregate properties of assemblies, such as base composition (expressed as GC content, GC%), tetranucleotide frequency spectra, and sequencing coverage (or read-depth) have been used previously to distinguish groups of contigs from metagenome assemblies, even with low-coverage Sanger sequencing, and thus infer the likely genetic composition of the genomes present in the sampled environment (Woyke et al. 2006). Full assembly of these constituent genomes is not usually attempted. The economics of second-generation sequencing permit generation of much higher coverage of the constituent organisms in a sample, and Mackelprang et al. (2011) obtained a 1.9 Mb draft genome of a methanogen from an environmental sample using Illumina sequencing. To achieve this, environmental shotgun reads were assembled and then contigs (contiguous sequences: fragments of genomes resulting from the assembly) separated using a combination of read coverage and tetranucleotide frequencies.

We have extended this approach to the analyses of symbioses, which may be regarded as low-complexity metagenomics problems. First, a preliminary assembly of all reads is made, and this is then analysed and visualised to identify the taxa present. The contigs and reads can then be separated by taxon, and finally each genome reassembled more stringently using diagnostic information obtained from the preliminary assembly. Here we demonstrate the methodology using raw genome sequence datasets for the *Dirofilaria immitis* (Nematoda) - *Wolbachia w*Di symbiosis, and for a dataset derived from an environmentally sourced *Caenorhabditis* species.

## Methods

### Sequencing

Standard Illumina genome sequencing protocols were followed. We extracted DNA from individual *D. immitis* female nematodes isolated from canine hosts and a sucrose- and detergent-cleaned plate culture of *Caenorhabditis sp. 5* nematodes using proteinase K and phenol-chloroform. For *D. immitis*, two paired-end libraries with insert sizes 110 bp and 340 bp were sequenced using 100 base and 76 base paired-end (PE) sequencing respectively. A long-insert (4 kb intended) mate-pair (MP) library was sequenced with 100 base PE reads. For *C. sp 5*, we generated two PE libraries with insert sizes 300 bp and 600 bp using 101 base PE sequencing. All 3′-end bases with a Phred quality below 20 were trimmed, and paired reads were discarded if either read in a pair was shorter than 35 bases. Reads from the long-insert MP library were trimmed to 50 bases to minimise the possibility of chimeric sequence. A total of 295 M reads totalling 28 Gb were generated for the *D. immitis* genome sequencing project (European Nucleotide Archive accession ERA032353) and 271 M reads totalling 17 Gb were obtained after quality trimming and filtering as above. For *C. sp 5*, 269 M reads totalling 26 Gb were obtained after quality trimming and filtering 282 M reads (28 Gb) of raw sequence data.

### Preliminary assembly

We used CLC Assembly Cell Version 3.2.2 (http://clcbio.com), with default parameters, to generate a preliminary assembly. No pairing information was used to scaffold initial contigs, and no coverage cutoff was utilised to filter contigs. CLC Assembly Cell returns all contigs greater than 200 bp in length.

### BLAST-based annotation of contigs

Ten thousand randomly selected contigs were queried against the NCBI nucleotide (nt) database using dc-megablast (McGinnis and Madden 2004) with an E-value cutoff of 1e-10. Using the NCBI taxonomy database (see http://www.ncbi.nlm.nih.gov/guide/taxonomy/) and a custom perl script, each contig was annotated with the taxonomic order of the best hit.

### Visualisation of GC%, coverage and taxonomic annotations

The GC% of each contig in the preliminary assembly was defined as the number of G or C bases divided by the length of the contig. The clc_ref_assemble_long alignment program from the CLC Assembly Cell suite was used to align reads to the preliminary assembly with default parameters. Alignments were post-processed to determine the average depth of coverage for each contig. A custom set of perl and R scripts was written to take GC%, coverage and taxonomic annotations and produce diagnostic visualisations. To reduce visual clutter, only taxa matching at least 10% of the annotated contigs were displayed. These scripts are freely available through http://nematodes.org/bioinformatics/blobology.

### Contig and read separation

The diagnostic information present in the GC%, coverage, and taxonomic annotation plot was used to inform a bespoke strategy for separating contigs belonging to different organisms. After identifying the taxa represented by each GC-coverage cluster of contigs, we created taxon-specific BLAST databases, and used these as filters to create sets of contigs, and ultimately reads, that were likely to derive from each genome of interest.

For *D. immitis*, the organisms to be separated were the nematode, its *Wolbachia w*Di and any canine host contamination. Canine contamination was detected by comparing to the dog genome sequence. We created two subsets of the NCBI nt database for the phylum Nematoda and for the class Alphaproteobacteria, and performed a BLASTn search of the preliminary assembly against both databases with an E-value cutoff of 1e-5. Using these BLASTn similarity data, we separated the preliminary contigs into four sets: *Nematoda*, *Alphaproteobacteria*, *Both*, and *Neither*. Contigs classified as *Both* were filtered and reclassified as *Nematoda* or *Alphaproteobacteria* if the best scoring hit to one database had a bit-score at least 50 more than the best hit to the other database. Contigs with hits to both databases but no clear difference between the hits (i.e. a bit-score difference less than 50) were classified as *Both*. Contigs that had no hits, or hits with a bit-score less than 50 were classified as *Neither*.

For *Caenorhabditis sp. 5*, where the presence of co-bionts was unexpected, a similar approach was used to identify nematode and bacterial contig sets.

### Stringent re-assembly of *Wolbachia w*Di

To assemble the *Wolbachia w*Di genome, we first retained all contigs classified as *Alphaproteobacteria*. We then discarded all contigs that were classified as *Nematoda*. We filtered the remaining contigs, classified as *Both* or *Neither*, based on the observed coverage of the *Alphaproteobacteria* contigs (which had a mean coverage of > 1000). Contigs with a low coverage (<250) were discarded. We extracted all read pairs where at least one read mapped best to one of these contigs to generate a highly *Wolbachia*-enriched raw sequence dataset. These putative *Wolbachia w*Di reads were reassembled using Velvet 1.1.05 (Zerbino and Birney 2008) using optimised parameter settings derived from the analysis of the preliminary assembly, and using the paired-end information ignored previously. The insert-size distribution of read-pairs was estimated more accurately by mapping the reads back to contigs in the preliminary SE assembly. We used VelvetOptimiser (VO) version 2.1.7 (http://bioinformatics.net.au/software.velvetoptimiser.shtml) to test k-mers from 41 to 63. VO picked 63 as the optimal k-mer and 360 as the expected k-mer coverage to get the longest contigs (k-mer coverage is lower than read coverage). The minimum k-mer coverage cutoff suggested by VO (5) appeared at odds with the expected k-mer coverage of 360, so we examined the coverage histograms as recommended in the Velvet manual, and picked 170 as an appropriate minimum k-mer coverage cutoff.

## Results

### Overview

Second-generation assembly algorithms that exploit the De Bruijn graph approach are powerful tools, but in order to deal with the many millions of reads that are typically involved in a genome project, several simplifying assumptions have to be made. In particular, it is assumed that all the data derive from one genome, and thus are, by definition, present in equimolar amounts and from one distribution of GC content. During assembly, contigs that appear to exceed these assumptions, for example sequences that derive from repeats, can be discarded to make the whole assembly problem computationally tractable. These assumptions are not met by many symbiotic systems, where one genome may be present at a very different effective molarity than others, and the different genomes may have different GC%.

**Fig. 1:**
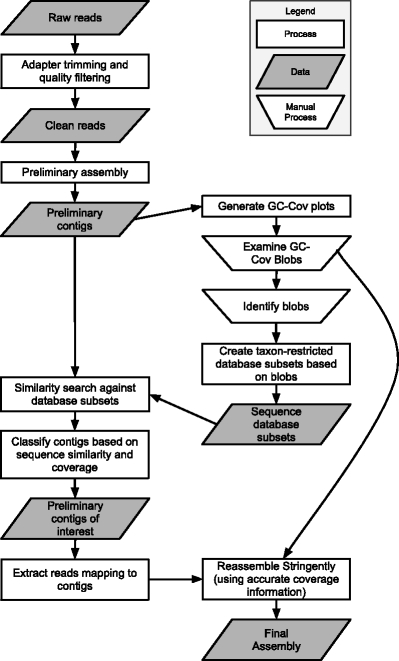
Pipeline for simultaneously sequencing symbiont and host genomes. To produce high-quality assemblies of the genomes of the nematode *Dirofilaria immitis* and its *Wolbachia w*Di bacterial endosymbiont we used a preliminary assembly to sort data into bins corresponding to each genome before performing high-stringency assembly on each separately

We developed a computational method to exploit the different properties of the several genomes present in symbiotic associations to firstly separate reads that were likely to derive from each genome into separate bins, and then carry out high-quality assemblies on each bin separately. Figure 1 provides an overview of our method. The key step is the preliminary assembly that acts as a diagnostic aid for assessing the composition of the sample and the coverage of the different organisms present in the sample. From this analysis, we then separate the contigs and reads into bins, corresponding to each genome present, and perform a high-stringency assembly using input parameters specific to each bin, such as insert size and coverage, derived from the preliminary assembly.

### *Dirofilaria immitis* and *Wolbachia* w*Di*: Diagnostic GC-coverage plots show organism-specific clusters

Initial attempts to assemble the *Wolbachia w*Di genome at the same time as the nuclear genome of the nematode *D. immitis* were partially successful, yielding 1.2 Mb of likely *Wolbachia* sequence across 259 scaffolds. The depth to which we expected to have sequenced this genome (in excess of 500-fold) led us to predict that a better assembly was achievable if the data could be better managed. In particular, we were concerned that chimaeric contigs might have resulted from illegitimate predictions involving *Wolbachia* genomic sequence and fragments of symbiont DNA inserted in the nematode nuclear genome.

We thus generated a preliminary assembly from 271 M quality-trimmed reads that resulted in 29,732 contigs spanning 84.2 Mb. The contig mean length and N50 (the contig length at which 50% of the total assembly length is contained in contigs of this length or greater) were quite low for a draft genome, at 2833 bp and 1581 bp respectively, as would be expected. We assessed the likely taxonomic origin of 10,000 randomly selected contigs using sequence similarity searches against nematode (host) and alphaproteobacterial (symbiont) databases. Only 3,490 contigs of the 10,000 contigs chosen at random from the initial assembly had a dc-megablast hit to the NCBI nt database at an e-value cutoff of 1e-10. No canine contamination was found.

**Fig. 2:**
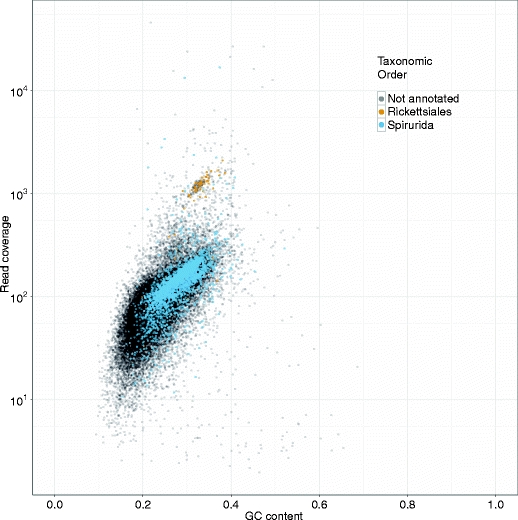
Taxon-annotated GC-coverage scatter-plot for a symbiotic system. The plot shows contigs from the *Dirofilaria immitis* - *w*Di preliminary SE assembly. Each dot represents one of 29,732 individual contigs. 10,000 contigs were randomly chosen for BLAST annotation, and 3,490 contigs with significant matches are coloured by putative taxon-of-origin. Full colour version is available online

We visualised these contig annotations in a scatter plot of GC% versus coverage, colouring the contigs by taxonomic annotation where this was available (Fig. 2). Two clusters of contigs were apparent: a large cluster with coverage between 20 and 300 and GC% between 15 and 35, and a much smaller, well-defined cluster with coverage greater than 1000 and GC% between 30 and 35. The large cluster was annotated with BLAST matches to nematodes from the order Spirurida (which contains the filarial nematodes such as *D. immitis*) whereas the smaller cluster was annotated with matches to the alphaproteobacterial order Rickettsiales (which includes *Wolbachia*). We thus identify the large cluster, with coverage ~150 fold, as largely comprising *D. immitis* contigs, and the small cluster, with coverage ~1000 fold, as *Wolbachia w*Di contigs. In this example therefore, coverage can be used to separate data deriving from the two genomes in the symbiotic association, while GC% cannot.

### Reassembly of *Wolbachia*-only reads improves *w*Di draft genome

Based on these analyses, we used a combination of sequence similarity searches and coverage (see Methods) to select 821 preliminary assembly contigs spanning 2.9 Mb. 23 M reads and their pairs (totalling 1.7 Gb of raw data; 0.6% of the initial filtered read set) mapped to these contigs and were extracted for an optimised assembly. These were reassembled stringently using Velvet, utilising expected and minimum coverage parameters, and also read pairing information.

We expected a genome size of ~1 Mb. The final assembly spanned 922,782 bp in only 14 scaffolds (Table 1), with a longest scaffold of 271,027 bp (i.e. more than 25% of the genome size) and a scaffold N50 of 219,165 bp. The assembly was annotated using RAST (Aziz et al. 2008) and 834 putative protein-coding open reading frames and 36 RNA genes identified (Table 1). Two of the short contigs, numbers 28 and 6, have a much higher than expected coverage and probably represent repetitive DNA. Contig 28 matches a previously sequenced ribosomal RNA from *w*Di. None of the other contigs less than 1000 bp long had any RAST features or hits to the Nematoda or Alphaproteobacteria subsets of the NCBI nt database, even with a sensitive BLASTn E-value cutoff of 1e-5. The majority of the *w*Di genome and all of the inferred protein-coding genes were present in only 9 scaffolds spanning 920,837 bp. Due to the stringent expected-coverage and coverage-cutoff parameters determined from the preliminary assembly, one can be confident that these scaffolds belong to the *Wolbachia* endosymbiont, and that they do not represent lateral gene transfer (LGT) events to the nematode host. LGT in the nematode host can be identified within the final assembly of the nematode genome at a lower coverage using the remaining reads and looking for stretches similar to the *Wolbachia* genome. A detailed analysis of the *Wolbachia w*Di genome is underway and will be published along with the genome of the host nematode *D. immitis.*

**Table 1:** The 14 scaffolds produced by Velvet on re-assembling putative *Wolbachia w*Di reads separately with stringent parameters

Velvet Assembly Contig ID	Length (bases)	k-mer coverage^a^	Annotated (RAST) features	Best blastn hit at 1e-5
8	271027	366.2	250	Alphaproteobacteria
27	219165	361.0	198	Alphaproteobacteria
4	126756	341.2	123	Alphaproteobacteria
18	86768	358.2	98	Alphaproteobacteria
3	76276	356.0	73	Alphaproteobacteria
5	56458	343.8	52	Alphaproteobacteria
10	44684	374.7	45	Alphaproteobacteria
1	39257	370.7	31	Alphaproteobacteria
28	1004	1705.9		Alphaproteobacteria
6	381	1938.6		
26	325	157.2		
87	258	182.4		
42	215	273.1		
13	208	246.0		

^a^Velvet reports ‘k-mer’ coverage rather than read coverage. The two are linearly related

### *Caenorhabditis sp. 5*: Unexpected co-bionts in a genome project

**Fig. 3:**
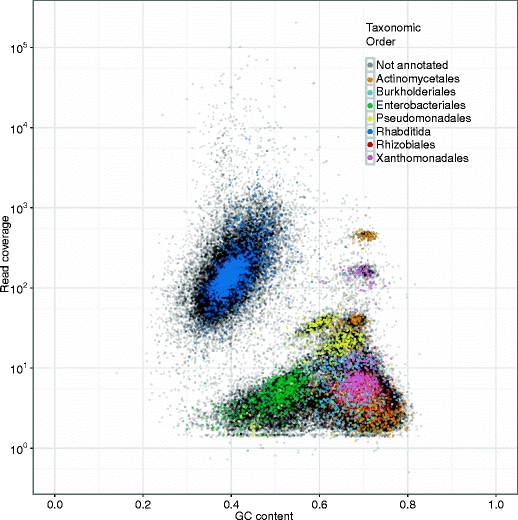
A low-complexity metagenome. Taxon-annotated GC-coverage scatter-plot (as in Fig. 2) for contigs from a preliminary SE assembly of a *Caenorhabditis sp 5* DNA sample. 10,000 randomly selected contigs were BLAST-annotated by comparison to the NCBI nt database, and coloured by class-of-origin of the best match identified. Full colour version is available online

In the *D. immitis* - *w*Di samples we were fortunate to have clean DNA that only included the nematode and its endosymbiont. However, when sampling organisms from the wild, or where association with co-bionts is unknown, the situation can be more complex. To demonstrate the utility of taxon-annotated GC-coverage plots for such samples, we generated a preliminary SE assembly and conducted GC%, coverage and taxonomic annotation analyses for a genome-sequencing project of the free living nematode *Caenorhabditis sp. 5* (Fig. 3). We had expected some bacterial contamination because the nematodes had been fed on *E. coli* but were surprised to see ten distinct clusters from seven taxonomic orders. Further analysis using various binning techniques (Kelley and Salzberg 2010; Parks et al. 2011; Saeed et al. 2011; Teeling et al. 2004) would be necessary for a full-scale metagenomic analysis. However, for our goal of obtaining a draft nematode genome, the taxon composition and coverage information gleaned from this diagnostic plot was enough to proceed with data binning and stringent re-assembly. We used the bacterial order identifications to create taxon-restricted databases and removed contigs (and associated read pairs) with high-confidence hits to these databases. We also used the mean coverage of the nematode cluster to discard very low-coverage contigs.

## Discussion

In this paper, we demonstrate a method to leverage the low cost and ultra high throughput of second-generation sequencing to access the genomes contributing to symbiotic associations. Previously, due to the cost of generation of raw data, it was operationally necessary to physically separate host and symbiont DNA before sequencing. Building on analyses of symbiont DNA in host-directed genome projects, and on the burgeoning field of metagenomics, we can view the metazoan host-bacterial symbiont(s) systems as simple metagenomics projects, and use computational tools to separate the various genomes involved after sequencing. This both reduces the cost of such projects and also makes genomic approaches feasible for systems where culturing or isolation of symbionts is not feasible.

Metagenome studies commonly perform the best assembly possible, using pairing and other information, and then explore GC%, coverage, sequence similarity and other sequence composition features to separate assembled contigs into clusters, but do not attempt subsequent reassembly. We propose that symbiosis researchers treat their systems like a reduced-complexity metagenome, but start by performing a preliminary assembly, ignoring pairing information, using all the short reads. It is then possible to explore GC%, coverage and taxonomic annotation of the contigs in the assembly, and devise bespoke filters to either collate all the reads likely to derive from the genome of interest, or remove those reads deriving from a contaminating (or host) genome. The project can then progress by stringently reassembling these reads using accurate pairing and coverage information obtained from the diagnostic GC-coverage plots.

The methodology is not exact, but generates tractable datasets and clear diagnostics to aid further experimentation. For example, we aligned the original reads back to the preliminary assembly to get an estimate of the read coverage for each contig. Although this method is not an accurate indicator of how the contigs were actually assembled from the reads (e.g. one can obtain short contigs with no reads aligned to them), it is sufficient for diagnostic purposes. Similarly, selecting a random subset of contigs for which to perform BLAST-based taxonomic annotation instead of the complete set has two advantages: the database search was faster, and the subset of annotated contigs provided an estimate of cluster density. Currently, we use the CLC Assembly Cell suite for the preliminary assembly and mapping because it is fast and has a low memory footprint. Other assemblers and mappers can be used equivalently. For the re-assembly step we prefer a coverage-aware genome assembler such as Velvet (Zerbino and Birney 2008; Zerbino et al. 2009) that can use coverage cutoffs and expected coverage information. The *Wolbachia w*Di endosymbiont draft genome was dramatically improved from 259 scaffolds to only 14 scaffolds following our method. To create a high quality genome with even fewer gaps, additional sequencing of long-insert DNA libraries would be needed, as with any other genome project.

Our method does not require an a priori assumption of the species composition of a sample and rapidly visualizes the different, sometimes unexpected, organisms present in DNA extractions from wild-caught or poorly-explored species. As demonstrated with the sequencing of the free living nematode *Caenorhabditis sp 5*, this method is useful not only for endosymbionts, but also for identifying contaminants, parasites, commensals, and co-bionts in cases where the DNA is obtained from an environmental sample that cannot be easily cultured or cleaned. We now create these plots for all our genome sequencing projects, whether or not we expect co-bionts, and highly recommend it as a quality-control step in any genome project.

LGT of genetic material from commensals and symbiont genomes into those of the host is a relatively rare but potentially evolutionarily significant process. While simple classification of contigs by best-BLAST match would risk conflating LGT fragments with their genome of origin, by utilising both coverage (resident LGT elements will display the coverage of their host genome) and GC% (LGT elements will tend to drift towards the GC% of their new host) as additional filters, it is possible to correctly partition contigs and the reads they represent by genome. Any mis-partitioned LGT read pairs will not co-assemble with the full genome of the taxon of origin, as they will have unique boundaries (and likely polymorphisms) compared to the resident orthologous sequence.

The *Wolbachia w*Di bacterial endosymbiont genome of the nematode *D. immitis* was assembled by computationally separating its reads from a mixed host and symbiont DNA sample. Without any additional cost for preparing purified bacterial cells and sequencing bacterial DNA, we can “buy one, get one free” while sequencing host metazoans. Researchers interested in symbionts, co-bionts or commensals can obtain draft genomes by collaborating with host genome projects through community websites such as arthropodgenomes.org and nematodegenomes.org (Kumar et al. 2011). The democratisation of sequencing initiated by the advent of next-generation technologies has already moved the locus of genome sequencing from large, industrial centres to many distributed laboratories. Similarly the kind of organisms being sequenced has shifted from inbred, laboratory-stock model species to wild, heterozygous and “contaminated” species. Approaches such as the one presented here will further democratise the process of genome sequence determination, and, *inter alia*, broker access to the biotic interactions between species, especially hosts and symbionts, that underpin biodiversity.

## References

[CR1] Aziz R, Bartels D, Best A, DeJongh M, Disz T, Edwards R (2008). The RAST Server: Rapid Annotations using Subsystems Technology. BMC Genomics.

[CR2] Dunning Hotopp J, Clark M, Oliveira D, Foster J, Fischer P, Muñoz Torres M (2007). Widespread lateral gene transfer from intracellular bacteria to multicellular eukaryotes. Science.

[CR3] Foster J, Ganatra M, Kamal I, Ware J, Makarova K, Ivanova N (2005). The Wolbachia genome of Brugia malayi: endosymbiont evolution within a human pathogenic nematode. PLoS Biol.

[CR4] Fuller C, Middendorf L, Benner S, Church G, Harris T, Huang X (2009). The challenges of sequencing by synthesis. Nat Biotech.

[CR5] Kelley D, Salzberg S (2010). Clustering metagenomic sequences with interpolated Markov models. BMC Bioinforma.

[CR6] Kumar S, Schiffer P, Blaxter M (2012) 959 Nematode Genomes: a semantic wiki for coordinating sequencing projects. Nucleic Acids Res 40(Database issue):D1295–1300 10.1093/nar/gkr826 PMC3245058 22058131

[CR7] Mackelprang R, Waldrop M, DeAngelis K, David M, Chavarria K, Blazewicz S (2011). Metagenomic analysis of a permafrost microbial community reveals a rapid response to thaw. Nature.

[CR8] McGinnis S, Madden T (2004). BLAST: at the core of a powerful and diverse set of sequence analysis tools. Nucleic Acids Res.

[CR9] Moran NA, Mira A (2001) The process of genome shrinkage in the obligate symbiont Buchnera aphidicola. Genome Biol 2:RESEARCH0054 10.1186/gb-2001-2-12-research0054 PMC64839 11790257

[CR10] Parks D, MacDonald N, Beiko R (2011). Classifying short genomic fragments from novel lineages using composition and homology. BMC Bioinforma.

[CR11] Pfarr KM, Hoerauf AM (2006). Antibiotics which target the Wolbachia endosymbionts of filarial parasites: a new strategy for control of filariasis and amelioration of pathology. Mini Rev Med Chem.

[CR12] Saeed I, Tang S-L, Halgamuge S (2011) Unsupervised discovery of microbial population structure within metagenomes using nucleotide base composition. Nucleic Acids Res. doi:10.1093/nar/gkr1204 10.1093/nar/gkr1204 PMC3300000 22180538

[CR13] Salzberg S, Dunning Hotopp J, Delcher A, Pop M, Smith D, Eisen M (2005). Serendipitous discovery of Wolbachia genomes in multiple Drosophila species. Genome Biol.

[CR14] Sinkins S, Walker T, Lynd A, Steven A, Makepeace B, Godfray (2005). Wolbachia variability and host effects on crossing type in Culex mosquitoes. Nature.

[CR15] Slatko B, Taylor M, Foster J (2010). The Wolbachi endosymbiont as an anti-filarial nematode target. Symbiosis.

[CR16] Teeling H, Meyerdierks A, Bauer M, Amann R, Glöckner F (2004). Application of tetranucleotide frequencies for the assignment of genomic fragments. Environ Microbiol.

[CR17] Wooley J, Godzik A, Friedberg I (2010). A Primer on Metagenomics. PLoS Comput Biol.

[CR18] Woyke T, Teeling H, Ivanova N, Huntemann M, Richter M, Gloeckner F (2006). Symbiosis insights through metagenomic analysis of a microbial consortium. Nature.

[CR19] Wu M, Sun L, Vamathevan J, Riegler M, Deboy R, Brownlie J (2004). Phylogenomics of the reproductive parasite Wolbachia pipientis wMel: a streamlined genome overrun by mobile genetic elements. PLoS Biol.

[CR20] Zerbino D, Birney E (2008). Velvet: algorithms for de novo short read assembly using de Bruijn graphs. Genome Res.

[CR21] Zerbino D, McEwen G, Margulies E, Birney E (2009). Pebble and rock band: heuristic resolution of repeats and scaffolding in the velvet short-read de novo assembler. PLoS One.

